# The Plant-Derived Compound Resveratrol in Brain Cancer: A Review

**DOI:** 10.3390/biom10010161

**Published:** 2020-01-19

**Authors:** Terezia Kiskova, Peter Kubatka, Dietrich Büsselberg, Monika Kassayova

**Affiliations:** 1 Department of Animal Physiology, Institute of Biology and Ecology, Faculty of Science, Pavol Jozef Safarik University, 04154 Kosice, Slovakia; terezia.kiskova@upjs.sk; 2 Department of Medical Biology and Biomedical Center Martin, Jessenius Faculty of Medicine, Comenius University in Bratislava, 03601 Martin, Slovakia; peter.kubatka@uniba.sk; 3 Department of Physiology and Biophysics, Weill Cornell Medicine in Qatar, Education City, Qatar Foundation, Doha 24144, Qatar; dib2015@qatar-med.cornell.edu

**Keywords:** resveratrol, brain cancer, glioblastoma, drug resistance

## Abstract

Despite intensive research, malignant brain tumors are among the most difficult to treat due to high resistance to conventional therapeutic approaches. High-grade malignant gliomas, including glioblastoma and anaplastic astrocytoma, are among the most devastating and rapidly growing cancers. Despite the ability of standard treatment agents to achieve therapeutic concentrations in the brain, malignant gliomas are often resistant to alkylating agents. Resveratrol is a plant polyphenol occurring in nuts, berries, grapes, and red wine. Resveratrol crosses the blood‒brain barrier and may influence the central nervous system. Moreover, it influences the enzyme isocitrate dehydrogenase and, more importantly, the resistance to standard treatment via various mechanisms, such as O6-methylguanine methyltransferase. This review summarizes the anticancer effects of resveratrol in various types of brain cancer. Several in vitro and in vivo studies have presented promising results; however, further clinical research is necessary to prove the therapeutic efficacy of resveratrol in brain cancer treatment.

## 1. Resveratrol

Resveratrol (RES) is a well-known polyphenol found in many plants, such as grapes (*Vitis vinifera*), mulberries (*Morus* sp.), and peanuts (*Arachis hypogaea*) [1]. It is a phytoalexin produced by spermatophytic plants in response to stress, injury, or UV radiation, or by fungal infection (e.g., *Botrytis cinerea*) and/or another pathogen [2,3,4]. The effects of different biotic and abiotic agents on the induced synthesis of RES in various plants have been studied. RES biosynthesis in plants occurs via the phenylalanine pathway [4]. The end product is synthesized as trans form, which may isomerize to cis form, or to trans and cis-piceid due to resveratrol 3-*O*-beta-glycosyltransferases [5]. Moreover, stilbene synthesis pathway is a side chain of the phenylpropanoid pathway, which may be considered an extension of the flavonoid pathway [6,7].

RES was first isolated in 1939 by Michio Takaoka from the root of *Veratrum grandiflorum* O. Loes [8]. In 1963, RES was defined as one of the chemical constituents of *Polygonum cuspidatum* (Ko-jo-kon) [9]. In 1976, the first reported detection method of trans-resveratrol has been described [10]. Thereafter, RES fell into oblivion until 1992, when Renaud and de Lorgeril described for the first time the “French paradox”—[11]. The “French paradox” is based on epidemiological data from French people who had a low incidence of coronary heart disease despite a high intake of dietary cholesterol and saturated fat. Actually, France is still a country with low coronary heart disease incidence and mortality when compared to the USA, UK, or Sweden [12,13,14]. During the same year, the concentration of RES in selected wines was measured [15]. In 2001, a study was carried out where the authors found an association between low to moderate wine intake and lower mortality from cardiovascular and cerebrovascular diseases [16]. After these observations, great attention has been paid to the French paradox and thousands of studies have been performed on various aspects of it [12].

### 1.1. Chemical Structure

**Figure 1:**
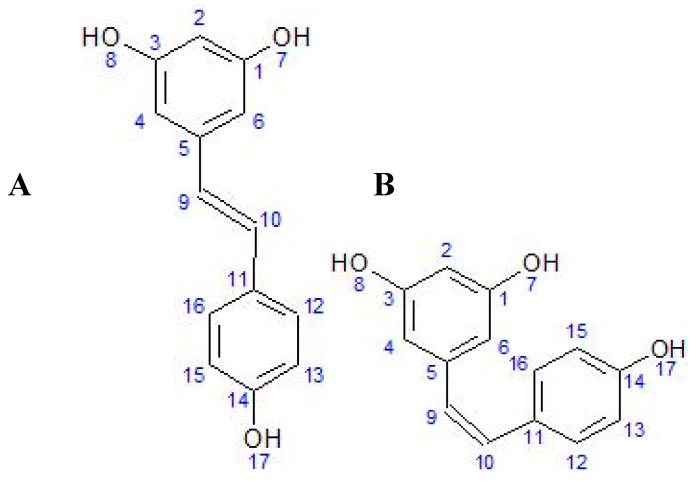
Chemical formula of trans (**A**) and cis (**B**) resveratrol (ACD/ChemSketch^®^).

Knowing the chemical structure of RES (3,4′,5-trihydroxy-trans-stilbene) is important for understanding its biological activity in living organisms. Due to the presence of more than one phenol group [17], RES belongs to the polyphenols [18]. RES is a stilbenoid polyphenol, possessing two phenol rings linked by an ethylene bridge [19]. Polyphenols often have antioxidant properties because they can react with free radicals and form a stable molecule that is less toxic than the radical itself [20,21]. Although the presence of a double ‘bridge’ makes it possible to form both the cis and trans forms of RES (Figure 1), the trans isomer is spatially more stable than the cis isomer [2]. Other minor conjugated forms containing 1–2 methyl groups (pterostilbene), a sulfate group (trans-resveratrol-3-sulfate), or a fatty acid have also been identified [22]. In spectrophotometric analysis, the maximum absorbance of trans-resveratrol is at approximately 304 nm, with cis-resveratrol at 286 nm [23]. The trans isomer is commercially available and converts to the cis form when exposed to UV radiation [24]. The stability of trans-resveratrol is influenced by various chemical and physical factors, e.g., light exposure, pH above 6.8, or temperature above 37 °C can cause degradation [25].

RES is an off-white powder (extracted by methanol), insoluble in water, but dissolves in ethanol or dimethylsulfoxide (DMSO) [26]. It has a melting point of 253‒255 °C and a molecular weight of 228.25 g/mol [2].

### 1.2. Metabolism of Resveratrol and Biotransformation

Glycosylation protects RES from oxidative degradation—glycosylated RES is more stable and soluble and readily absorbed in the human gastrointestinal tract [27]. The metabolism of RES is a complex process, involving various pathways, predominantly the conjugation to glucuronides and sulfates in phase II. Both isomers of RES undergo glucuronidation by uridine-diphosphate-glucuronosyltransferase (UGT) to two corresponding glucuronides, 3′-*O*-glucuronide and 4′-*O*-glucuronide [22], accounting for its predominant urine excretion [28]. Moreover, in humans, RES is subject to sulfate conjugation by sulfotransferases to form resveratrol-3′-*O*-sulfate and resveratrol-4′-*O*-sulfate [22,29]. Abundant trans-resveratrol-3-*O*-glucuronide and trans-resveratrol-3-sulfate were identified in rat urine, mouse serum, and incubations with rat and human hepatocytes [30]. Several other sulfate conjugates (4′-sulfate, 3,5-disulfate, 3,4′-disulfate, 3,4′, 5-trisulfate) have been identified in the rat [22].

Some RES metabolites are derived from intestinal bacterial metabolism. Dihydroresveratrol was also later identified in rat urine [31], plasma [32], and, most importantly, in mammalian fecal bacterial species [33]. These RES conjugates are then either absorbed by the intestine or excreted in the urine. Up to 50% of the RES dose can be metabolized in this way [34]. 

### 1.3. Accumulation of Resveratrol in Tissues

After entering into an organism, the RES in plasma reaches a half-life of 8–14 min; however, for its metabolites it is around 9.2 h [28]. RES binds to some proteins and protein transporters in the blood stream, to serum albumin or to lipoproteins in the order high-density lipoprotein (HDL) < low-density lipoprotein (LDL) < very low-density lipoprotein (VLDL) [35,36]. The absorption of RES occurs by passive diffusion [37] or by transport via ion channels [38] to pass through the cell membrane, allowing its intracellular biological action [39,40]. 

After oral administration, RES is absorbed [41] and accumulates in various organs, such as the stomach [42], intestines, or liver [28,30,42,43,44], as sites of its extensive absorption and metabolism [45]. RES (and its metabolites) is able to accumulate in target cells or organs of various diseases [46] including cancer, such as breast cancer tissue [47,48] and colorectal [49,50] or leukemia cancer cells [51]. RES and its metabolites accumulate in myocardial tissue [52] and even in the ocular tissues after oral administration [53]. However, no RES accumulation in the tumor tissue of neuroblastoma in athymic mice was observed [54].

### 1.4. Bioavailability of Resveratrol and Potential Side Effects

Over the past few decades, RES has received widespread attention as a preventive agent for numerous diseases. However, low bioavailability limits its use. After oral administration in humans, up to 75% of RES is absorbed, possibly by transepithelial diffusion [34]. However, oral bioavailability is low (<1%) due to rapid and extensive metabolism in the intestine and liver [55,56,57]. Thus, increasing the bioavailability is one of the aims nowadays. As has been shown, when loaded in casein nanoparticles, the oral availability of RES increased up to 10 times [58]. Various methodological approaches have been developed in recent years. These include several delivery systems such as the encapsulation of RES in lipid nanocarriers or liposomes, emulsions, micelles, insertion into polymeric nanoparticles, solid dispersions, and nanocrystals [59,60]. For example, the bioavailability of RES from the grapevine shoot extract Vineatrol30 has been significantly increased using a liquid micellar formulation, without any treatment-related adverse effects, making it a suitable system for improved supplementation [61]. On the other hand, the bioavailability of RES delivered through oral mucosa may be significantly higher than by swallowing, as determined by the fraction of the initial RES intake in the blood and, under metabolized form, in the urine [62].

Numerous studies described various side effects of RES [19,63,64]. In a single high dose (500 mg) of RES in 15 healthy volunteers under fasting conditions, no side effects were seen after 24 h [57]. However, long-term administration of RES at a dose of 2.5 g per day led to diarrhea, vomiting, or nausea in healthy volunteers [65]. Interestingly, no severe side effects were reported during long-term administration (up to one year) of doses of up to 16 mg grape RES [66]. Also, renal toxicity has been reported after a dose of 5 g RES in the form of SRT501 (developed by Sirtris, a GSK company) in two cycles during multiple myeloma, but no renal toxicity was observed in healthy controls, type 2 diabetics, or patients with mitochondrial encephalomyopathy, lactic acidosis, and stroke-like episodes (MELAS) syndrome [67]. The results suggest that the right dose of RES is essential to target specific diseases. 

### 1.5. Biological Effects of Resveratrol

It has been shown that RES possesses numerous therapeutic effects, such as antioxidant [68,69,70,71], anti-inflammatory [72,73,74,75], cardioprotective [76,77,78,79], or analgesic effects [80,81,82], and has an impact on diabetes and obesity [83,84,85]. RES has been further studied for its increasing relevance in various neurological disorders, such as Alzheimer’s [86,87,88], Parkinson’s [89,90], and other neurodegenerative diseases [91,92], as well as brain tumors [93,94,95]. In addition, RES showed anticancer activity in many other cancer types, such as breast, prostate, skin, lung, liver, or colorectal cancer, as reviewed, for example, in [45,96,97,98]. 

### 1.6. The Passing of Resveratrol through the Blood‒Brain Barrier

**Figure 2:**
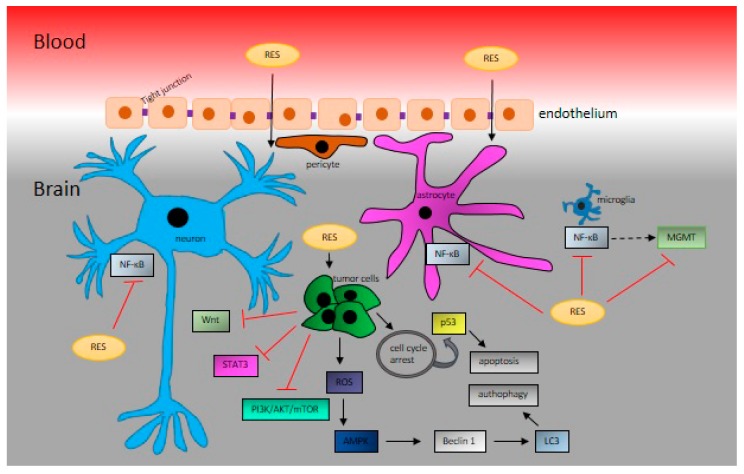
Resveratrol (RES) crosses the blood‒brain barrier via tight junctions [104]. In the brain, RES inhibits NF-κB in neurons, astrocytes, and microglia. In brain cancer cells, RES exerts proapoptotic activities via influencing the cell cycle. RES induces oxidative stress, leading to autophagy. Moreover, RES influences cancer cells via various signaling mechanisms, including the PI3K/AKT/mTOR pathway, Wnt, or STAT3.

In 2002, Sinha et al. showed that RES exerts protective effects against oxidative stress in middle cerebral artery occlusion model stroke in rats [99]; however, they did not monitor the ability of RES to cross the blood‒brain barrier (BBB). Nonetheless, during the same year, it was shown that RES crosses the BBB successfully and thus may protect against global cerebral ischemic injury [100]. One of the fundamental pathophysiology changes during ischemia reperfusion injury is the collapse of the BBB. RES at a dose of 50 mg/kg of body weight significantly decreased the infarct volume and improved the neurological score 24 h after reperfusion. Moreover, it improved the balance of matrix metalloproteinase-9 (MMP-9) and its endogenous inhibitor, TIMP-1. Thus, RES attenuated cerebral ischemia by maintaining the integrity of BBB via the regulation of MMP-9 and TIMP-1 [101]. At a lower dose of 20 mg/kg of body weight, RES reduced the cerebral infarct size, and improved BBB breakdown via the Hippo/YAP/TAZ pathway [102]. In another model of BBB dysfunction, autoimmune encephalomyelitis (EAE), RES at doses of 25 and 50 mg/kg of body weight was dose-dependently able to decrease EAE paralysis, ameliorate EAE-induced loss of tight junction proteins ZO-1, occludin, and claudin-5, and repress the EAE-induced increase in adhesion proteins ICAM-1 and VCAM-1 [103]. In addition, RES suppressed the EAE-induced overexpression of proinflammatory transcripts iNOS and IL-1β and upregulated the expression of anti-inflammatory transcripts arginase 1 and IL-10 cytokine in the brain, downregulated the overexpressed NOX2 and NOX4 in the brain, and suppressed NADPH activity [103]. However, the functional relationship between RES, BBB, brain cancer development, and antitumor therapy has not yet been determined (Figure 2).

## 2. Resveratrol in Brain Cancer Studies

In Europe, the incidence of primary CNS cancers ranges from 4.5 to 11.2 cases per 100,000 men and from 1.6 to 8.5 per 100,000 women. Astrocytic tumors include aggressive phenotype tumors such as glioblastoma (GBM). The five-year survival of primary brain cancers varied from 4.9% for high-grade to 43% for low-grade tumors [105]. GBM accounts for approximately 65% of all primary brain tumors and is characterized by low survival, with only 10% of patients surviving for five years [106]. GBM is one of the most malignant types of central nervous system tumors. Despite advances in treatment modalities, it remains largely incurable [107]. Gliomas account for almost 80% of all primary malignant brain tumors [108]. These include astrocytic tumors (astrocytoma, anaplastic astrocytoma and GBM), oligodendrogliomas, ependymomas, and mixed gliomas [109]. Despite the variety of modern therapies against GBM, it is still a deadly disease with extremely poor prognosis. Patients usually have a median survival of approximately 14 to 15 months from diagnosis [109,110].

The current gold standard in the treatment of GBM is temozolomide (TMZ)—an oral alkylating agent. TMZ is known to induce cell cycle arrest at G2/M, which leads to apoptosis. The cytotoxicity of TMZ is mediated by its addition of methyl groups at N7 and O6 sites on guanines and the O3 site on adenines in genomic DNA. Alkylation of the O6 site on guanine leads to the insertion of a thymine instead of a cytosine opposite the methylguanine during subsequent DNA replication, and this can result in cell death [111]. When TMZ is given concomitantly with radiotherapy, a statistically prolonged patient survival compared to TMZ-only therapy was shown (26.5% vs. 10.4% of the two-year survival rate). On the other hand, the concomitant treatment with radiotherapy plus TMZ resulted in grade 3 or 4 hematologic toxic effects in up to 10% of patients [112]. However, at least 50% of TMZ-treated patients do not respond to TMZ. This is due primarily to the overexpression of O6-methylguanine methyltransferase (MGMT) and/or a decreased rate of DNA repair in GBM cells [111,113,114], involving a critical regulator of the p53 tumor suppressor, an MDM2 protein. MDM2 is overexpressed in many human malignancies. It inhibits DNA break repair [115]. Another mechanism of resistance of human gliomas causes ATP-binding cassette (ABC) transporters to be overexpressed by the endothelial and/or epithelial cells of the BBB and the blood‒tumor barrier [116]. 

One common feature in brain cancer types is the mutated form of isocitrate dehydrogenases (IDHs). NAD (+)-dependent IDHs in the mitochondria play a pivotal role in the production of NADH from NAD+ in the Krebs cycle. As reviewed before, IDH mutations inhibit glioma stem cells’ differentiation by producing high levels of 2-hydroxyglutaric acid (2-HG), regulate vascular endothelial growth factor (VEGF) to promote the formation of the tumor microenvironment, and induce high levels of hypoxia-inducible factor-1α (HIF-1α) to promote glioma invasion [117]. IDH mutations also repress the tumor-associated immune system by inhibiting complement activation, while reducing the number of tumor-infiltrating T cells, phagocytosis and the excretion of cytokines. The oncometabolite 2-HG also affects epigenetics and genome stability. So, there are clinical trials being conducted on inhibitors of mutant IDH1, which target the production of 2-HG [118]. IDH1 mutations were predominantly found in human low-grade astrocytoma, oligodendroglioma, and secondary GBM. On the other hand, IDH2 mutations occurred less frequently in gliomas and were mutually exclusive of IDH1 mutations [119]. RES has been found to maintain IDH levels in a middle cerebral artery occlusion stroke model [120] and to protect the left ventricle by increasing IDH activity in myocardial infarction [121]. It has also been shown that RES stimulated a mitochondrial Complex I decrease in NADH concomitant with increased IDH levels in liver cells [122]. Even if many studies are dealing with the effect of RES on the Krebs cycle and mitochondrial enzymes, no study has described the direct potential of RES action on IDH during brain cancer.

### 2.1. The Effect of Various Routes of Administration

As described previously, RES has low bioavailability. However, the anticancer action of RES can be slightly modified by various routes of administration. Protection of RES from extensive metabolization in the gastrointestinal tract and liver increases its bioavailability [123], which is especially important in intracranial malignancies. RES administration via oral gavage or ad libitum in the water supply suppressed subcutaneous GBM xenograft growth in mice; intratumor or peritumor RES injection had a more pronounced effect on tumor volume [94]. RES administration via lumbar puncture effectively inhibited the growth of intracranial orthotopic rat GBM and prolonged the mean survival time of tumor-bearing animals [124]. Furthermore, a wide distribution of apoptotic foci with decreased Cyclin D1 staining, as well as enhanced autophagy with upregulated autophagy-related protein LC3 and Beclin 1, was found after RES treatment in brain tumor tissue [124,125]. Lumbar puncture is even more effective than intra-arterial RES administration. Shu et al. [125] demonstrated a 5-fold higher concentration of RES in the whole brain after lumbar puncture compared to intra-arterial external carotid artery injection. Additionally, combination therapy such as lumbar-punctured RES with neurosurgery significantly improved the prognosis of rats with advanced orthotopic GBM, prolonged the postoperative survival time, suppressed tumor growth, induced apoptosis, and inactivated STAT3 signaling [126].

During brain cancer treatment, targeted RES delivery to the brain tumor tissue could help to overcome the low bioavailability, poor water solubility, and chemical instability of RES. To improve GBM treatment, various types of liposomes and polymeric nanoparticles were developed. Vijayakumar et al. [127] reported that the biological half-life, passive brain targeting, and antiglioma cytotoxicity of RES were significantly enhanced by using d-α-tocopheryl polyethylene glycol 1000 succinate (TPGS)-coated liposomes (RES-TPGS-Lipo). Guo et al. [128] modified the surface of RES-loaded polyethylene glycol‒polylactic acid nanoparticles with transferrin moieties (Tf-NP-RES), which led to increased intracellular uptake, higher cytotoxicity, and apoptosis of rat C6 and human U-87 MG GBM cell lines in vitro compared to free RES and nanoparticles without transferrin. Since transferrin receptors are exclusively expressed in brain capillaries [129], the accumulation of Tf-NP-RES in tumor tissue, decreased tumor volume, and prolonged survival were shown in rats bearing C6 orthotopic glioma. Similar results were obtained in the subcutaneous xenograft U-87 MG mouse model. Moreover, S-phase cell cycle arrest, activation of caspases 3/7, and higher production of reactive oxygen species were demonstrated in vitro [130]. Sallem et al. [131] have designed a new nanovector system for the delivery of a synthetic derivative of the RES molecule to the brain tissue, based on superparamagnetic iron oxide nanoparticles. This nanohybrid did not affect the mitochondrial metabolism, but damaged the plasma membrane of C6 glioma cells in vitro, indicating cytotoxic effects. The in vivo activity of this system still needs to be elucidated. Furthermore, the antitumor efficacy of RES-loaded nanoparticles may be enhanced by combination with other food-derived natural polyphenols, where synergistic effects are expected. Mukherjee et al. [132] have prepared liposomal TriCurin (TrLp; curcumin: epicatechin gallate: RES 4:1:12.5) and demonstrated that TrLp upregulates the activated protein p53 in cultured mouse GBM cells in vitro. Additionally, TrLp caused repolarization of M2-like tumor-associated microglia/macrophages to the tumoricidal M1-like phenotype, led the intratumoral recruitment of activated natural killer cells, suppressed tumor growth, and promoted the apoptosis of GBM and GBM stem cells in vivo [132]. Neves et al. [133] used solid lipid nanoparticles functionalized with apolipoprotein E, leading to increased (1.8-fold higher) permeability through the hCMED/D3 monolayer. 

### 2.2. Resveratrol and Standard Anticancer Therapy

Numerous studies have shown that RES is able to alleviate the side effects induced by chemotherapeutic drugs [98,134]. Moreover, in combination with other anticancer agents, RES synergistically or additively enhances their efficacy against various types of cancer [135]. RES can reverse multidrug resistance and also can act as a sensitizer of cancer cells to standard chemotherapeutic drugs [97]. It has been demonstrated that RES increases TMZ efficacy through several mechanisms. TMZ induces both apoptosis and autophagy in human glioma cells through a reactive oxygen species (ROS) burst and extracellular signal-regulated kinase (ERK) activation. However, during these processes, autophagy protects glioma cells from apoptotic cell death. RES has been shown to augment the therapeutic capacity of TMZ by reducing ROS/ERK-mediated autophagy and subsequently increasing apoptosis both in vitro and in vivo [136]. In the human SHG44 GBM cell line, the combination RES+TMZ displayed additive antiproliferative effects by increased ROS production, subsequent activation of AMPK, inhibition of mTOR signaling, and downregulation of antiapoptotic protein Bcl-2. These results were confirmed in the orthotopic xenograft mouse model as the reduction of tumor volume and decreased expression of Ki-67, a marker of proliferation [137]. GBM-initiating cells (GICs) display stem cell properties and play a pivotal role in tumor development, resistance to TMZ, and tumor recurrence [138]. RES enhanced the sensitivity of these highly resistant cells to TMZ via activation of the DNA double strands/pATM/pATR/p53 pathway, leading to the activation of apoptosis. Additionally, RES promoted the differentiation of GIC involving p-STAT3 inactivation [139]. A RES dimer, ε-viniferin, has been shown to augment the apoptosis of the GBM cell line induced by another chemotherapeutic agent, cisplatin, under in vitro conditions through the activation of caspases 3, 8, and 9 [140]. 

RES also acts as a radiosensitizing anticancer agent in the prostate, skin, colon, breast cancer, hepatoma, leukemia, and others [141], including brain malignancies [142,143,144]. It has been demonstrated that RES is a radiation sensitizer for highly radioresistant human SU-2 glioma stem cells. The synergistic effect of RES and radiation was seen in the inhibition of cell proliferation, induction of autophagy, promotion of apoptosis, prevention of DNA repair in the early stage, and induction of differentiation, both in vitro and in vivo [145].

### 2.3. Antiproliferative and Proapoptotic Effects of Resveratrol

Antiproliferative, proapoptotic, and anti-inflammatory activities are considered to be the most important anticancer mechanisms of RES in different types of tumors [96,146,147]. Mammalian cell proliferation comprises two processes: (a) the cell cycle, including duplication of genetic material and cell division; (b) cell growth, regulated by many growth factors. The four phases of the cell cycle, i.e., G1 (Gap 1), S (synthesis), G2 (Gap 2), and M (mitosis), are mainly regulated by cyclin-dependent kinases (CDKs) that act in a complex with their cyclin partners [148]. Cell cycle arrest is an irreversible process that can result in apoptotic cell death [147]. RES was able to delay the cell cycle progression and inhibited the proliferation of rat C6 glioma cells by arresting the cell cycle at S phase at micromolar concentrations [145]. The authors demonstrated the inhibition of the expression of specific oncogenic microRNAs (miRs) such as miR-21, miR-19, and miR-30a-5p in glioma cells, which was consequently associated with altered expression of their targeting genes such as p53, STAT3, EGFR, COX-2, NF-κB, and the PI3K/AKT/mTOR signaling pathway. Moreover, RES suppressed tumor growth and prolonged survival of rats bearing intracranial C6 glioma [145]. Induction of S-G2/M cell cycle arrest by RES was also described in human GBM cell lines and was accompanied by an increase in levels of pCdc2(Y15), cyclin A, E, and B and a decrease of cyclin D1 [149]. A recent investigation by Laaniste et al. [150] revealed that, in low-grade gliomas, the M2 gene-regulatory network, consisting of 177 genes and governing G2 to M progression, is substantially and significantly downregulated by RES. Transcription of late cell cycle genes such as FosM1 and B-Myb was the most affected, even at nanomolar RES concentrations [150].

Deregulation of precursor cell differentiation plays a crucial role in brain tumor development. Therefore, differentiation-promoting agents may potentially suppress GBM and medulloblastoma growth, reduce tumor resistance, and prevent recurrence in patients [151]. In human U87MG cells, RES induced glial-like and neuronal-like differentiation, as evidenced by decreased expression of nestin (stem cell marker) and, on the other hand, by increased expression of a glial acidic fibrillary protein (a mature glial cell marker) and of beta III-tubulin (a neuronal differentiation marker) in a time-dependent manner [152].

Some studies have indicated that RES also displays its anticancer activity on the level of posttranscriptional regulation of gene expression. Tristetraprolin (TTP) is an RNA binding protein that can bind AU-rich elements in target mRNAs with high affinity and then promote the deadenylation and decay of target transcripts such as proto-oncogenes, antiapoptotic genes, immune regulatory genes, and others [153,154]. In U87MG human glioma cells, RES increased TTP expression, thereby inducing apoptosis and suppressing cell growth [155].

### 2.4. Resveratrol and Proteins of Resistance in Brain Cancer

Though TMZ-based chemotherapy following neurosurgery has been proven to be effective, not all patients benefit clinically because of TMZ resistance. The most important feature of TMZ resistance is the expression of the protein MGMT [115]. It has been shown that RES reverses the TMZ-induced resistance of T98G GBM cells by downregulation of MGMT by the NF-κB-dependent pathway [156]. Repression of the activated Wnt signaling pathway through the downregulation of MGMT expression seems to be another way of inhibiting proliferation and facilitating the apoptosis of resistant glioma cells by the combination RES+TMZ [157]. The presence of RES forced various GBM cells (U87-MG, U-138 MG, and U251) treated with TMZ through mitosis leading to mitotic catastrophe and senescence, reducing the clonogenic capacity of cells and increasing the chronic effects of TMZ [158]. 

Another mechanism of resistance provide ABC transporters that are overexpressed in the BBB [116]. It has been previously reviewed that RES is able to reverse multidrug resistance via various mechanisms [49,159]. However, no study deals with this mechanism directly in brain cancer.

### 2.5. Resveratrol and Cellular Senescence

Cellular senescence is an irreversible cell cycle arrest that is considered to be an important tumor-suppressive mechanism as it stops proliferation. Therapy-induced senescence is thought to be an effective tool in cancer treatment, with fewer side effects than apoptosis-inducing treatment [160,161]. In a study with U87 and U118 human glioma cell lines, RES inhibited proliferation by inducing cellular senescence in a dose- and time-dependent manner [162]. RES induced significant changes in cell volume and cell morphology: spindle-shaped glioma cells were transformed to hypertrophic, flat cells expressing senescence-associated-β-galactosidase, a marker of senescence [161]. Moreover, RES inhibited the mono-ubiquitination of histone H2B at K120 (uH2B) [162]. Another study has shown that RES-induced senescence of human and rat glioma cells was increased by the inhibition of histone deacetylases [163]. The role of the histone deacetylase sirtuin 2 (SIRT2) as a mediator of the inhibitory action of RES on GBM stem cell (GSC) proliferation was revealed by Sayd et al. [164]. The blockade of the GSC cell cycle by RES at doses lesser than 150 μM was mediated by SIRT2, whereas GSC necrosis induced by higher doses of RES was independent of sirtuin activity. Yang et al. [165] have shown that RES-induced glioma cell senescence, apoptosis, and antiproliferative effects could also be mediated by downregulation of POK erythroid myeloid ontogenic factor (Pokemon), at least partially through enhancement of the recruitment of histone deacetylase 1 (HDAC1) [165].

### 2.6. Resveratrol and STAT3 Signaling

Signal transducer and activator of transcription (STAT) 3 is a member of the family of transcription factors that is involved in the transmission of extracellular signals into the nucleus, thereby influencing the transcription of various genes. In carcinogenesis, STAT3 upregulates genes that can promote tumor survival, angiogenesis, resistance to cell death, and cell cycle progression [166]. Upregulation of STAT3 in GBM has been demonstrated in numerous studies, as reviewed by Kim et al. [167]. STAT3 is required for tumor formation and maintenance of the self-renewal of GBM stem-like cells [168,169], some of which express CD133 as a cancer stem cell marker [170]. It has been shown that RES displays its anticancer action on GBM-CD133+ tumor-initiating cells by inhibition of cell growth and viability, induction of apoptosis, suppression of self-renewal capacity, and enhancement of radiosensitivity in vitro and in vivo through the suppression of the STAT3 pathway [144]. Furthermore, in medulloblastoma, the most common type of primary brain malignancy in children [171], RES suppressed cell growth by STAT3 downregulation, decreased the incidence of STAT3 nuclear translocation, and promoted neuronal differentiation of medulloblastoma cells by axon regeneration and accumulation of SOCS3 to the synapse-like end of long cell processes [172]. 

### 2.7. Resveratrol and p53

TP53 is a tumor suppressor protein commonly known as “a guardian of the genome”. TP53 is one of the most commonly deregulated genes in various tumors, including GBM [173,174]. Suppression of p53 activity is associated with the activation of a serine/threonine protein kinase AKT, thereby promoting the survival and proliferation of tumor cells [175]. RES reduced AKT phosphorylation and induced p53 expression and subsequent transcription of downstream p53 target genes such as Bax, Pig8, and TP53INP in GBM cells. These changes led to the inhibition of cell growth and invasion of U87 glioma cells and glioma stem-like cells as well as the suppression of GBM mouse xenograft growth [94]. Moreover, in A172 and T98G GBM cell lines with a heterozygous p53 mutation, RES has been able to restore wild-type p53 expression via the activation of intracellular Notch-1 expression in a time-dependent manner. Simultaneous dephosphorylation of AKT, increased Bax expression, decreased Bcl-2 expression, and cleavage of caspase-3 were observed in this study, suggesting strong proapoptotic action of RES [176]. In a population of patient-derived glioma stem cells, which are responsible for tumor progression and poor patient prognosis, RES was found to reduce the self-renewal and tumor-initiating capacity of these cells via activation of the p53/p21 pathway and degradation of Nanog, a transcription factor essential for the retention of stemness [177]. 

### 2.8. Resveratrol and Wnt Signaling

As mentioned above, RES enhances the antiglioma efficacy of TMZ by inhibiting the Wnt signaling pathway, both in vitro and in vivo [157]. It has been demonstrated that the Wnt signal is essential for the self-renewal, migration, and differentiation of GBM stem cells [178]. The study of Cilibrasi et al. [93] revealed the highly heterogenous response of seven stem cell lines isolated from GBM-suffering patients to RES exposure. RES generally modulated the Wnt system, inhibited cell proliferation, increased cell mortality, and strongly decreased cell motility and invasiveness. As a result, suppression of nuclear β-catenin levels, increased transcription activity of Wnt target gene MYC, and a drastic decrease of c-Myc protein were observed. Additionally, RES inhibited epithelial‒mesenchymal transition through downregulation of transcription factors Twist1 and Snail1 [93].

## 3. Conclusions and Future Directions

Resveratrol, a plant polyphenol occurring in nuts, berries, grapes, and red wine, demonstrates well-described anti-inflammatory, anti-oxidative, cardioprotective, and analgesic properties. Resveratrol is a molecule with very low toxicity that targets multiple molecular signaling pathways and consequently affects numerous carcinogenesis-related genes. The significant antineoplastic potential of resveratrol was demonstrated in many cancer types when administered alone or in combination with diverse anticancer agents and targeted therapies. It is known that resveratrol crosses the blood‒brain barrier and influences the brain’s structure. Its ability to prevent carcinogenesis in the brain includes the suppression of oxidative stress and inflammation, as well as inhibition of cell proliferation with the triggering of cell death mechanisms. It may influence cancer cells’ activity via affecting various signaling mechanisms, including NF-κB, p53, Wnt, PI3K/AKT/mTOR, or STAT3. It has been described that resveratrol alleviates the resistance to standard alkylating agents, such as temozolomide, through influencing O6-methylguanine methyltransferase. However, the interaction between resveratrol and mutated isocitrate dehydrogenase, one of the key features of various brain cancer types, needs to be elucidated. Several other important questions must be resolved before the introduction of resveratrol into the clinical management of brain malignancies. To the best of our knowledge, no clinical trials have been conducted yet to evaluate the efficacy of resveratrol against brain cancer in humans. However, due to the number and complexity of cancer-related signaling pathways affected by resveratrol, further investigations are needed to overcome its pharmacokinetic limitations such as poor bioavailability in humans, describe its precise anticancer mechanisms of action, and define its efficacy in different brain cancer cell subtypes. With the intention to increase resveratrol’s bioavailability in organisms and its potential as an adjuvant drug in clinical oncology, research should focus on resveratrol’s delivery systems, formulations, dosing protocols, modulations of cancer cell metabolism, and possible interactions with other anticancer drugs. Finally, the development of optimized analogs of the resveratrol molecule to fit the specific mechanisms of anticancer action and increased stability and bioavailability in organisms should lead to its improved anticancer activity and reasonable clinical use.

## 4. Data Search Strategy

Data from the English-language biomedical literature were analyzed from the PubMed bibliographic database using terms such as “resveratrol”, “brain cancer”, “chemical properties”, “metabolism”, “bioavailability”, “side effects”, “blood‒brain barrier”, “glioblastoma”, and “medulloblastoma” as a keyword or medical subject heading (MeSH) term. We focused on in vitro, in vivo, and clinical studies published from 2013 to 2020. As no clinical trials were found in PubMed, data were also reviewed from the U.S. National Institutes of Health database (http://www.clinicaltrials.gov/).

## Abbreviations

2-HG	2-hydroxyglutaric acid
ABC	ATP-binding cassette
AKT	Protein kinase B
COX	Cyclooxygenase
DMSO	Dimethylsulfoxide
EAE	Autoimmune encephalomyelitis
EGFR	Epidermal growth factor receptor
ERK	Extracellular signal-regulated kinase
GBM	Glioblastoma
GSC	Glioblastoma stem cells
HDL	High-density lipoprotein
HIF-1α	Hypoxia inducible factor-1α
ICAM	Intercellular adhesion molecule 1
IDH	Isocitrate dehydrogenase
LDL	Low-density lipoprotein
MELAS	Mitochondrial encephalomyopathy, lactic acidosis, and stroke-like episodes syndrome
MGMT	O6-methylguanine methyltransferase
MMP	Matrix metalloproteinase
mTOR	Mammalian target of rapamycin
NADPH	Nicotinamide adenine dinucleotide phosphate
NOX	NADPH oxidase
PI3K	Phosphoinositide-3-kinase
Pokemon	POK erythroid myeloid ontogenic factor
RES	Resveratrol
ROS	Reactive oxygen species
SIRT	Sirtuin
STAT	Signal transducer and activator of transcription
TAZ	Transcriptional coactivator with PDZ-binding motif
TIMP	Tissue inhibitor of metalloproteinases
TMZ	Temozolomide
TTP	Tristetraprolin
UGT	Uridine-diphosphate-glucuronosyltransferase
VCAM	Vascular cell adhesion molecule
VEGF	Vascular endothelial growth factor
VLDL	Very low-density lipoprotein
YAP	Yes-associated protein
